# Pulmonary vein occlusion and lung infarction complicating non-treated moderate single pulmonary vein stenosis after radiofrequency ablation of atrial fibrillation

**DOI:** 10.1259/bjrcr.20160091

**Published:** 2017-03-18

**Authors:** Khalid M Alfudhili, Hesham H Hassan, Hesham Abdullah, Mohsen Sherbiny

**Affiliations:** ^1^Department of Radiodiagnostic and Interventional Radiology, Dammam Medical Complex, Dammam, Saudi Arabia; ^2^Cardiology Department , Saud Al-Babtain Cardiac Center, Dammam Medical Complex, Dammam, Saudi Arabia

## Abstract

Pulmonary vein (PV) radiofrequency ablation (RFA) is an effective, curative technique for selected group of patients with atrial fibrillation (AF) refractory to antiarrhythmic drugs. However, pulmonary vein stenosis (PVS) is a potential complication which may present clinically as non-specific respiratory symptoms that often under-recognized or misdiagnosed leading to progression of low-grade stenosis to complete occlusion if not treated with timely intervention.

## Summary

Catheter-guided percutaneous radiofrequency ablation RFA is a relatively new effective therapy for drug-refractory atrial fibrillation AF. The ectopic foci of abnormal electrical activity in the ostia or muscular sleeves of the pulmonary veins are the target of ablation.^1^ Nowadays, although it is relatively uncommon complication due to improved experience and change of ablation strategy, PVS may occur following this procedure.^1–3^ Mild and moderate stenosis are usually asymptomatic. On the other hand, haemodynamically significant high-grade PVS or occlusion may lead to localized pulmonary venous hypertension or infarction, resulting in a diversity of respiratory symptoms.^3,4^ Owing to non-specific nature of these symptoms and imaging findings in cases of severe stenosis or occlusion, pulmonary venous infarction could be easily misdiagnosed as parenchymal disease or infection. Importantly, asymptomatic moderate or severe PVS may progress rapidly to complete occlusion and result in pulmonary hypertension, pulmonary infarction and recurrent respiratory infections.^5^ We present this case report to describe misleading pulmonary parenchymal findings associated with single pulmonary vein occlusion (PVO) complicating non-treated moderate PVS.

## Case report

**Figure 1.**
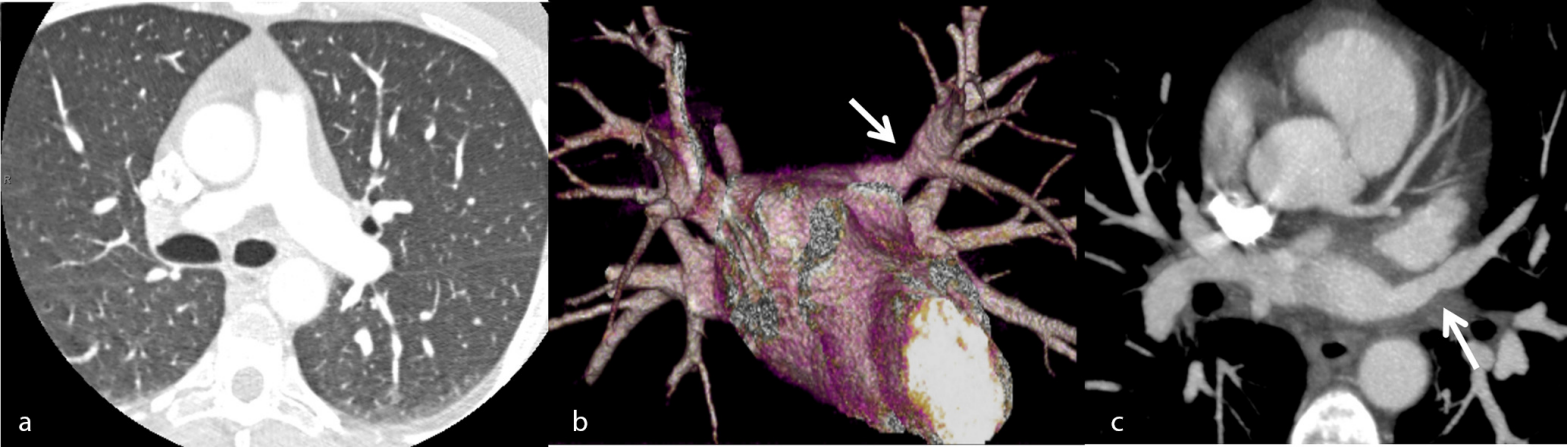
Three months post-ablation follow-up cardiac CT scan: (a) Lung window shows normal pulmonary parenchyma. (b and c) 3D reconstruction of left atrium and mediastinal window enhanced CT axial section respectively show left upper pulmonary vein LSPV moderate stenosis (arrows).

A 35-year-old male patient, with persistent AF refractory to the pharmacologic treatment underwent RFA in our hospital. As a routine practice in our centre, the patient was assessed pre-procedurally by transesophageal echocardiography (TEE) on the day of procedure to delineate anatomy of pulmonary veins and left atrium (LA) and to exclude LA appendage thrombus. During RFA procedure all four PV were individually isolated under guidance of intracardiac echocardiography. RF energy was delivered using a conventional 4 mm and 8 mm tip ablation catheters and a power setting of 30 W, 50 °C around a circular decapolar catheter located at the pulmonary veins ostia. Application of the RF was immediately interrupted when microbubbles were detected by the intracardiac echocardiography (ICE). After a curative ablation therapy patient was kept on aspirin to prevent pulmonary venous or arterial thrombosis and recurrence of AF.^3^ Ambulatory follow-up cardiac computed tomography (CT) angiogram was done 3 months following RFA and showed approximately 60% left superior PVS but no intervention was performed because the patient was asymptomatic and he was kept on oral anticoagulation (Figure 1).

Two months later, the patient developed cough associated with hemoptysis, mild intermittent fever, mild left-sided pleuritic chest pain and shortness of breath on exertion. He visited the primary health centre close to his residency where chest radiography was done and showed left upper lobe poorly marginated opacities and minimal left pleural effusion. Further laboratory studies were done to rule out pulmonary tuberculosis (TB) which were within normal limits. A definitive diagnosis was not made and the patient was given broad spectrum antibiotics and referred to pulmonology clinic in our hospital for further investigation.

**Figure 2.**
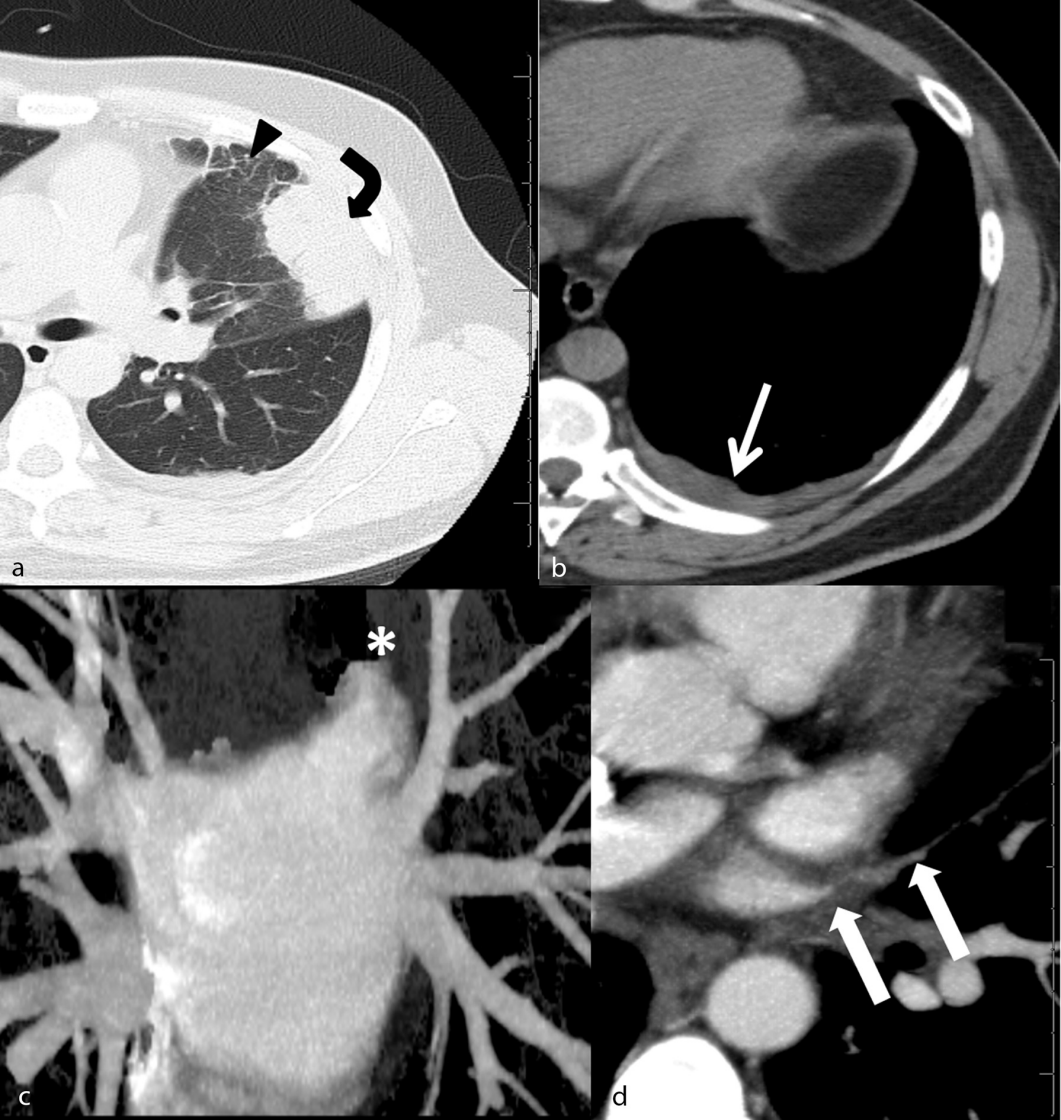
(a and b) Non-enhanced chest CT scan: lung window (a) shows pleural-based parenchymal consolidation (curved arrow) associated with adjacent subtle ground glass attenuation and interlobular septal thickening (arrow head). Mediastinal window (b) shows left-sided minimal pleural effusion (thin arrow). These pulmonary findings are related to total LSPVO revealed by (c) 3D reconstruction of left atrium (asterisk), note other pulmonary veins patency. (d) Contrast-enhanced CT scan axial section depicts total occlusion of LSPV.

Patient presented to our hospital 6 months after ablation without improvement in the symptoms. Non-enhanced CT was done and showed increased parenchymal attenuation and multiple peripheral patchy consolidations in the apico-posterior and anterior segments of left upper lobe associated with minimal left pleural effusion (Figure 2a and b). Cryptogenic organizing pneumonia (COP), chronic eosinophilic pneumonia (CEP), fungal infection (pulmonary aspergillosis), lung cancer and primary pulmonary lymphoma were suggested as differential diagnosis and ultrasound-guided aspiration of the left-sided pleural effusion was carried out and the obtained specimen was fluid of serous nature. Besides that, lung biopsy was also performed and the histopathologic examination revealed surprisingly intimal hyperplasia associated with multifocal haemorrhagic infarction due to PVO and hypertensive pulmonary arteriopathy. After that contrast-enhanced CT scan was performed with 3D reconstruction and manifested clearly an occluded left superior pulmonary vein (LSPV) (Figure 2c and d). Occlusion was confirmed by conventional angiography. Ventilation/perfusion scan demonstrated absent perfusion of the involved lung parenchyma and left upper lobectomy was warranted.

## Treatment

Unfortunately, the patient was treated by left upper lobectomy because of several reasons including the relatively long time period between the procedure and diagnosis of PV stenosis (more than 6 months), evidence of complete occlusion of the vein on CT angiogram and at cardiac catheterization, and complete absence of left upper lobe perfusion at ventilation–perfusion scintigraphy.

## Outcome and follow-up

Patient had no complaints at his regular follow-up visits. No new abnormality was detected on physical examination or radiological tests.

## Discussion

AF is one of the most common forms of abnormal cardiac rhythm that represents a major cause of stroke.^6^ The prevalence of AF increases with age reaching up to 5% in people older than 65 years. Over 90% of ectopic beats that provoke AF arise in the pulmonary veins, nearly 50% of them originate in LSPV.^1,6,7^

Catheter-based RFA is an effective therapy that has been recently performed with increasing frequency for symptomatic patients with refractory AF not responding to antiarrhythmic pharmacological therapy.^8^ Around 76% of the patients who underwent RFA for AF remain symptom-free for a period of 1 year.^7^ Nevertheless, this procedure is not free of risks and one of the potential complications encountered following the ablation is stenosis of one or more of pulmonary veins.^4–6^ The prevalence of this complication is still a contentious issue in the medical literature, with reported prevalence ranging from 1.5–42% depending on the technique of ablation and the diagnostic method used for diagnosis of venous stenosis.^3,6,8^ Early stenosis of PV can develop in initial days following the ablation as a result of tissue swelling caused by thermal necrotic injury of the venous wall which may either subside gradually or progress to scarring and constriction.^6^ Di Biase et al reported in a large consecutive series that the duration between RFA to the initial diagnosis of PVS by CT varied between the first 3 months up to 25 months. In addition, 18 of 1780 patients involved in this series were found to have total PVO, the most severe form of PVS after RFA, with mean time for development of total occlusion of at least one PV was 9.9 months (1–30 months).^5^ From a clinical point of view, the time period between RFA to the onset of respiratory symptoms for patients could also be variable. Packer et al reported in their series of 23 patients that these patients developed symptoms over the course of 103 ± 100 days of follow-up.^3^

Symptoms of PVS/PVO depend on many factors such as the number of veins involved, the severity of stenosis and the time course of disease. Almost all patients with mild (< 50%) or moderate PVS (50–70%) are asymptomatic.^4,5^ Of interest, clinical presentation of severe stenosis (> 70%) and total occlusion may include cough, dyspnea on exertion, pleuritic chest pain, hemoptysis and recurrent respiratory infections.^3,9^ Due to the variability in symptomology which typically suggest lung pathology, patients may be referred to pulmonologists instead of cardiologists. Consequently, lung parenchymal abnormality caused by clinically significant PVS /PVO may be misdiagnosed erroneously as recurrent bronchopneumonia, granulomatous disease such as pulmonary TB, pulmonary aspergillosis, interstitial lung disease, CEP, COP, pulmonary thromboembolism or primary or secondary lung malignant lesion leading to numerous unnecessary investigations and potentially dangerous inappropriate management.^2,6^ Nevertheless, the wide range of differential diagnosis of pulmonary parenchymal abnormality seen on CT scan and chest radiography can be shortened if the radiologist takes into consideration if the abnormality is acute or chronic, and the radiologic findings viewed in conjunction with not only the radiological appearance but also with clinical situation of the patient. For instance, semi-invasive or chronic necrotizing aspergillosis usually seen in patients with debilitating illness and radiological manifestations include segmental consolidation which may be bilateral with cavitation and pleural thickening. Furthermore, the characteristic appearance of CEP on chest radiograph and CT scan images is bilateral peripheral consolidations and usually associated with peripheral blood eosinophilia. Peripheral and peribronchovascular patchy consolidation is the main radiological finding of COP but the most two radiologic characteristics helpful in diagnosis of COP are the migrating nature of the consolidations and the “reverse halo” or atoll sign.^10,11^

Accurate pre-procedural cardiac assessment provides an important information regarding the PV and LA prior to AF ablation which includes: (1) the number of PV, (2) PV ostial caliber, (3) measurement of LA dimensions, (4) presence of LA appendage thrombus and (5) identification of anatomic anomalies (6) illustrate the anatomy of the interatrial septum which may help guide trans-septal puncture and (7) provide anatomic knowledge of structures such as the esophagus to minimize post procedure complications. ICE, TEE, electrocardiogram (ECG)-gated CT and Magnetic resonance imaging (MRI) have become an increasingly important tool in the pre-procedural assessment of these patients. Because TEE is suboptimal for proper atrio-venous junction evaluation and PV ostial diameter estimation, ECG-gated CT scan and MRI of the LA and pulmonary veins are currently the most appropriate techniques used in many centres with which the morphology and size of pulmonary veins can be delineated, and LA thrombus is excluded before ablation procedures.^12^ Generally, the mean ostial diameter of the pulmonary veins as measured at computed tomography CT is variable as follows: right superior, 11.4–12.4 mm; left superior, 9.6–10.5 mm; right inferior, 12.3–13.1 mm and left inferior, 9.0–9.9 mm. These measurements can serve as a guideline when interpreting CT and MRI examinations to identify possible PVS.^13^

Post-RFA radiological assessment of the presence and the degree of severity of PVS is extremely important. Many radiological modalities have been investigated to evaluate mediastinal PVS such as plain radiography, ventilation/perfusion (V/P) scan, TEE, conventional pulmonary venography, CT scan and MRI. Chest radiography usually depict non-specific peripheral parenchymal opacity with small unilateral pleural effusion and atelectasis. V/P scintigraphy may show a decreased or absence of perfusion and a normal or diminished ventilation pattern in one lobe or whole lung. TEE is often limited to image deeply into all four pulmonary veins particularly the superior veins and it is less useful in demonstrating the location and extent of PVS. However, it may reveal elevated pulmonary pressure, increased PV flow velocity and turbulence in the LA. Conventional pulmonary venography may show directly PV stenosis/occlusion, pruning of peripheral pulmonary arteries and delayed transit time through the lungs to the LA.^6,9^

CT scan and to a lesser extent MRI are currently the best tests used for evaluation of PVS because of several advantages such as noninvasiveness and visualization of the adjacent mediastinal structures. MRI offers other advantages including freedom from ionizing radiation and providing information about blood flow as well as right and left ventricular function. On the other hand, contrast-enhanced CT venography is not only useful for revealing of PVS or PVO directly but also useful for showing indirect morphological alterations in the pulmonary parenchyma due to functional consequences of a haemodynamically significant stenosis or occlusion, obtaining 3D reconstruction easily and visualizing of the veno-atrial junctions with excellent spatial resolution.^2,3,6,9^ CT scan findings which may be seen in the lung parenchyma and mediastinum include multifocal peripheral consolidations or nodular lesions, interstitial septal thickening, ground-glass (mosaic) attenuation, hilar localized lymphadenopathy, pleural thickening, pleural effusion, increased attenuation of the mediastinal fat adjacent to the site of stenosis due to fibrosis and broncho-vascular ectasia.^6,9^

To our knowledge, there is general agreement that symptomatic severe stenosis of one or multiple pulmonary veins should be considered for restoration of pulmonary flow by catheter-guided balloon angioplasty with different opinions about stent implantation as an initial therapy of clinically significant PVS.^2^ Nevertheless, controversy exists on whether intervention is required for asymptomatic patients with severe single PVS. Indeed, because of the delay in PV angioplasty in some patients, corresponding lung perfusion may not increase after restoration of PV patency. Therefore, Saad et al and Di Biase et al suggested that early PV interventional angioplasty in such patients may normalize lung perfusion and lead to prevention of associated pulmonary parenchymal disease.^4,5^ Furthermore, restenosis rate after PV dilatation is relatively high (30–50%) even with stent placement. Hence, patients treated with angioplasty should be carefully monitored for recurrent respiratory symptoms and followed with CT scan to document restenosis. Prompt treatment should be performed when an important restenosis is identified due to possible rapid progression to total occlusion.^2,9^

Our patient was initially diagnosed with pulmonary TB based on clinical presentation and chest radiograph. Subsequently, as non-enhanced chest CT findings (namely peripheral pleural-based parenchymal consolidations, subtle interlobular septal thickening, ground glass attenuation and pleural effusion) seen in the left lung were similar to those described in cases of COP, CEP, pulmonary semi-invasive aspergillosis, bronchioloalveolar carcinoma and primary pulmonary lymphoma, these diseases were suggested as possible diagnosis. However, pulmonary veno-occlusive disease was not considered in the differential diagnosis as the history of RFA was not emphasized when the CT study was originally requested. Given the potentiality of rapid progression of asymptomatic PVS to symptomatic complete occlusion within 1–3 months of initial development,^2^ pulmonary angioplasty was worthwhile to be done for our patient after the 3-month follow-up CT which showed moderate left superior PVS (around 60%) to normalize blood flow to the corresponding lung and preserve pulmonary function. In other words, this delay in the interventional treatment for an established moderate PVS and misinterpretation of symptomatology and radiological findings of PVO led to unnecessary investigations and delay of the appropriate timely management.

## Learning points

1. A high degree of suspicion is required to promptly diagnose PVS, as diagnostic tests can be misleading. In other words, imaging findings of sequential changes involving lung parenchyma in a patient presents with respiratory symptoms and a previous history of RFA, haemodynamically important PVS should be considered as a diagnosis after excluding infection, interstitial lung disease and thromboembolism.
2. Although patients with post-ablation moderate stenosis of a single pulmonary vein are often asymptomatic, these patients are at increased risk of progression to complete PVO with or without pulmonary infarction. Accordingly, physicians should be highly vigilant for the development of symptoms of PVS in this patients group.^5^
3. Early interventional treatment of asymptomatic moderate single PV stenosis should be considered to prevent progression to complete occlusion or thrombosis and normalize of pulmonary perfusion and function.
4. After the initial baseline post- ablation CT scan, follow-up should include careful monitoring for positive symptoms with prompt CT evaluation if symptoms related to PVS/PVO occurred. Alternatively, even if the patient is asymptomatic, serial follow-up CT scan may be also performed at 3-month intervals or earlier if the patient developed new symptoms suggestive of PVS/PVO with keeping in mind the radiation dose.^5^

## Consent

Written informed consent for the case to be published (including images, case history and data) was obtained from the patient for publication of this case report, including accompanying images and is within the hospital records.
